# Transfer learning assessment of small datasets relating manufacturing parameters with electrochemical energy cell component properties

**DOI:** 10.1038/s44334-025-00024-1

**Published:** 2025-04-18

**Authors:** Francisco Fernandez, Soorya Saravanan, Rashen Lou Omongos, Javier F. Troncoso, Diego E. Galvez-Aranda, Alejandro A. Franco

**Affiliations:** 1 Laboratoire de Réactivité et de Chimie des Solides, UMR CNRS 7314, Université de Picardie Jules Verne, 80039 Amiens Cedex, France; 2 Réseau sur le Stockage Electrochimique de l´Energie (RS2E), FR CNRS 3459, Hub de l’Energie, 15 rue Baudelocque, 80039 Amiens Cedex, France; 3 ALISTORE-European Research Institute, FR CNRS 3104, Hub de l’Energie, 15 rue Baudelocque, 80039 Amiens Cedex, France; 4 Institut Universitaire de France, 1 rue Descartes, 75231 Paris Cedex 05, France

**Keywords:** Method development, Batteries, Fuel cells

## Abstract

The performance of electrochemical cells for energy storage and conversion can be improved by optimizing their manufacturing processes. This can be time-consuming and costly with the traditional trial-and-error approaches. Machine Learning (ML) models can help to overcome these obstacles. In academic research laboratories, manufacturing dataset sizes can be small, while ML models typically require large amounts of data. In this work, we propose a simple but still novel application of a Transfer Learning (TL) approach to address these manufacturing problems with a small amount of data. We have tested this approach with pre-existing experimental and stochastically generated datasets. These datasets consisted of component properties (e.g., electrode density) related to different manufacturing parameters (e.g., solid content, comma gap, coating speed). We have demonstrated the robustness of our TL approach for manufacturing problems by achieving excellent prediction performance for electrodes in lithium-ion batteries and gas diffusion layers in fuel cells.

## Introduction

Electrochemical energy cells (EECs) are devices that work through redox (reduction-oxidation) reactions. These include energy storage devices, such as Lithium-Ion Batteries (LIBs) cells, and energy conversion devices, such as Proton Exchange Membrane Fuel Cells (PEMFCs). Both types of devices play a crucial role in the green transition needed to mitigate climate change. Due to the intermittent nature of renewable energy sources and the need for on-demand electricity generation, EECs can facilitate more effective integration of these energy sources into the energy matrix. On one side, LIBs offer several key advantages, including high energy density, extended lifespan, and low self-discharge. These benefits have had a considerable impact on various industries, including electronics and telecommunications, making them essential components in portable devices^1,2^. LIBs are also contributing to the electrification of the transportation industry by being used in electric vehicles (EVs)^3^. On the other side, PEMFCs also present several advantages, such as high efficiency, low operating temperature, and zero carbon emissions^4^. They present a viable alternative for applications where EVs face limitations, such as heavy-duty trucks. However, despite the mentioned advantages, both LIBs and PEMFCs face challenges that require further research and development efforts to optimize their manufacturing processes to achieve better performance and efficiency^5,6^.

The redox reactions in a commercial LIB cell occur at its main components, the positive and the negative electrodes. The final electrochemical performance of these components depends on their microstructure, which is highly influenced by the manufacturing process parameters^7^. The manufacturing process includes different stages. The first of them is the slurry preparation, which consists of mixing Active Material (AM), electron conductive additive, binder, and solvent. This is followed by the slurry coating over the current collector. Then, since this is a wet process, it is followed by the drying step, where the solvent is evaporated to get the dried electrode. Then, this dried electrode is calendered to improve the contact between the electrode and the current collector. In addition, this step reduces the overall thickness, which improves the cell energy density. By controlling the manufacturing parameters in each one of these steps, like the AM chemistry selected for the slurry preparation or the gap and the speed of the rolls during calendering, one is able to also control the electrode microstructure. Therefore, it is essential to understand the effect of these manufacturing parameters on the final electrode properties (density, porosity, mass loading, tortuosity factor) to choose the best set of parameters that allow the optimization of the LIB cell performance^8–10^.

For PEMFCs, the Gas Diffusion Layer (GDL) is one of the most important components that controls the electrical and thermal conduction. It also influences the reactant gases’ dispersion, diffusion, and water management, which in turn affects the performance of the PEMFC. The three main stages of the GDL manufacturing involve the carbon fiber production, followed by the preparation of the carbon paper substrate and finally, the finishing treatment. This process starts with a wet-spinning step, in which polyacrylonitrile-derived carbon fibers are processed into precursor fibers. These carbon fibers are then sized and chopped after stabilization and carbonization. In order to improve the mechanical stability, conductivity, and achieve the desired porosity, the next step is to mix the previous carbon fibers with water and binder. These are then subjected to papermaking, bonding, impregnation, curing, and carbonization steps. Finally, the GDL substrate is dipped in polytetrafluoroethylene (PTFE). By varying the manufacturing parameters involved in the mentioned steps, such as the weight percentage of carbon and PTFE loading, the properties of the GDL can be changed, such as electronic and thermal conductivities, porosity and geometric tortuosity^11,12^. This manufacturing process is also relevant for optimizing the GDL performance.

For many years, numerous studies based on experimental approaches have been conducted to gain insight into the electrochemical, physical, and mechanical phenomena in LIB electrodes and PEMFC GDLs^13–23^. The objective is to elucidate the relationship between input factors and output properties^23^. However, designing experiments is a complex process due to the large number of input parameters and the intrinsic properties of the materials involved. This hinders the optimization of the LIB electrodes and PEMFC GDLs manufacturing processes^24,25^. Additionally, experimental outputs have limited resolution, and often, it is difficult to characterize and track the time series of the microstructure evolution. This limits the way researchers can control and optimize experiments. To compensate the intrinsic experimental limitations, computer simulations appear as efficient complementary tools to study LIB electrodes and PEMFC GDLs. Computational modeling allows to understand the underlying process by numerically solving the mathematical equations describing the physical process. These techniques have also contributed to evaluating LIB electrodes and PEMFC GDLs processes and improving their performance^26–29^.

In the case of LIB cell manufacturing, the ARTISTIC project^30^ has been a pioneer in optimizing these processes with different computational modeling techniques at the mesoscale. The ARTISTIC project has proposed 3D-resolved dynamic simulations of the entire manufacturing chain of electrodes and cells, from the slurry preparation, passing by the drying, the calendering of the resulting electrode, the electrolyte filling of the electrodes and cells, and the resulting cell performance. These physics-based computational models are designed to simulate and predict the influence of materials properties and manufacturing parameters on the electrode and cell properties^31^. They have been calibrated and validated against experimental data. To face the computational cost that these models can have, data-driven Machine Learning (ML) surrogate models have been trained with the physics-based models obtained datasets to accelerate the optimization of different manufacturing steps^24,25,32,33^. For example, a Deep Learning (DL) model has been trained to track microstructure evolution over time during the electrode calendering step by using Discrete Element Method (DEM) time series data^34^. Also, in the PEMFC field, ML models have been trained to predict various properties of the GDLs as a function of their most relevant manufacturing parameters^35^.

Given the numerous variables that impact the final component quality and performance, it is not straightforward to optimize the manufacturing processes using a trial-and-error method. In this scenario, ML techniques are excellent tools for unveiling patterns and relationships hidden in manufacturing datasets. They also allow for faster optimization of the parameters involved in the manufacturing steps^25,36^. ML algorithms have a significant potential in other critical domains. These include applications such as material discovery, real-time monitoring, state estimation, battery usage, fault detection, and life cycle management^37^. As we have previously demonstrated in our ARTISTIC project initiative, ML can be used to accelerate both the understanding of the processability of new materials (chemistry and formulation), and the optimization of LIB electrode and cell manufacturing process^38^.

Supervised ML techniques require labeled data (where each input data point has an associated output value) to enable data-driven predictions and optimizations. In contrast, unsupervised ML uses unlabeled data to identify patterns within it. For LIB cells, both mentioned types of ML have been applied to experimental datasets. Specifically, for LiNi_0.33_Mn_0.33_Co_0.33_O_2_ (NMC) electrodes, we can find studies involving different stages of the manufacturing process with various ML methods. For instance, Pinto-Cunha et al. analyzed Decision Trees (DTs), Support Vector Machine (SVM), and Deep Neural Networks (DNNs) to find the interdependencies between slurry parameters and NMC final properties^39^. Duquesnoy et al., in addition to considering slurry parameters, included coating parameters to develop an automatic methodology which used a Gaussian Naives Bayes classifier to assess the homogeneity or heterogeneity of the resulting electrode^40^. ML was also used to predict the influence of electrode formulation and calendering conditions on the electrode properties^41^. Furthermore, K-Means clustering was used in the calendering step of NMC-based electrodes by Primo et al.^42^. Regarding graphite anodes, Faraji-Niri et al. collected a lab-scale dataset containing control variables in the slurry and coating stages, that were used to train Random Forest (RF) models for predicting final properties of the electrode^43^. For the State of Health (SoH) estimation of commercial LGM50 cells, Faraji-Niri et al. trained a Gaussian Process Regressor by selecting features from electrochemical impedance spectroscopy data^44^. Moreover, Neural Networks (NNs) have been developed for the detection of cracks in LIB electrodes by using 3D image data^45^ and for mapping the 3D architecture of NMC particles with focused ion beam slicing in sequence with electron backscatter diffraction data^46^. Meanwhile, for GDLs, various literature works show the use of ML models for different applications. For instance, Hou et al. formulated an Extreme Learning Machine model that allowed to determine the optimal GDL structure parameters with the minimum temperature, maximum current density, and good oxygen concentration uniformity^47^. Shum et al. used DT and Convolutional NN (CNN) algorithms to segment GDL’s x-ray computed tomography image stacks, comparing their performance with basic image processing techniques^48^. In addition, Cawte et al. developed a 3D CNN to predict the GDL materials’ permeability directly from 3D binary image data^49^. Froning et al. also employed a CNN model to predict the GDL materials’ permeability, but with stochastically generated microstructures^50^. Saco et al. tested and observed SVM regression, Linear Regression (LR) and k-Nearest Neighbors algorithms on different humidification processes during experimental studies of PEMFCs^51^. They concluded that, in their case of study, LR provided better accuracy than other models^51^. Furthermore, for deformed GDL, Wang et al. built an M^5^ model, which included multi-physics and multi-phase flow simulation, ML-based surrogate modeling, multi-variable and multi-objects optimization. This M^5^ model proved to be effective and efficient for optimizing the GDL current density and oxygen distribution^52^. It is worth noting that all these previous works have emphasized the development of ML tools for analyzing vast datasets. Surprisingly, none of the previous works addressed the problem of how to derive reliable ML models with smaller datasets, either due to the lack of sufficient experiments or the computational cost of running large simulations to produce synthetic data.

Transfer Learning (TL) is a very interesting paradigm within ML that uses the knowledge learnt from solving one task to accelerate learning and improve performance on a related but different task. This approach uses an existing ML model trained on a vast dataset in the source domain and adapts or extends it to extrapolate its predictive power to another domain with a smaller target dataset. This significantly reduces training time and data requirements compared to building a model from scratch. TL practices are typically associated with NNs, which allow data-driven strategies to benefit from existing knowledge in related domains, improving their overall performance. TL has demonstrated excellent results in various fields, such as medicine, mechanics, art, physics, security, or biology^53^. Specially, it has shown interesting results for tasks involving computer vision^54,55^ and natural language processing techniques^56,57^. TL has also been used in LIB and PEMFC research, particularly for cell SoH estimations and aging prediction, extrapolating predictions to different domains and usage conditions^58–62^. As far as we know, TL has not been used in LIB electrodes or PEMFC GDLs manufacturing at the time of this article writing.

There are four main approaches for transferring knowledge: feature-based, instance-based, parameter/model-based, and relational/adversarial-based^53,63^. By adjusting the weights of individual instances (data points), instance-based transfer helps to bridge the gap between the source and target domains, even when the overall data distributions are different. Feature-based transfer focuses on transferring the feature representation learned from the source domain to the target domain, while parameter-based transfer directly transfers the weights and biases learned by the pre-trained model as a starting point for the target domain model. In NN models, this approach freezes some layers and/or finetunes some layers, and/or adds some new layers to the original model. Finally, relational-based TL approaches transfer logical relationships or rules learned from the source domain to the target domain and are usually associated with generative adversarial networks^54–59,63^.

**Fig. 1:**
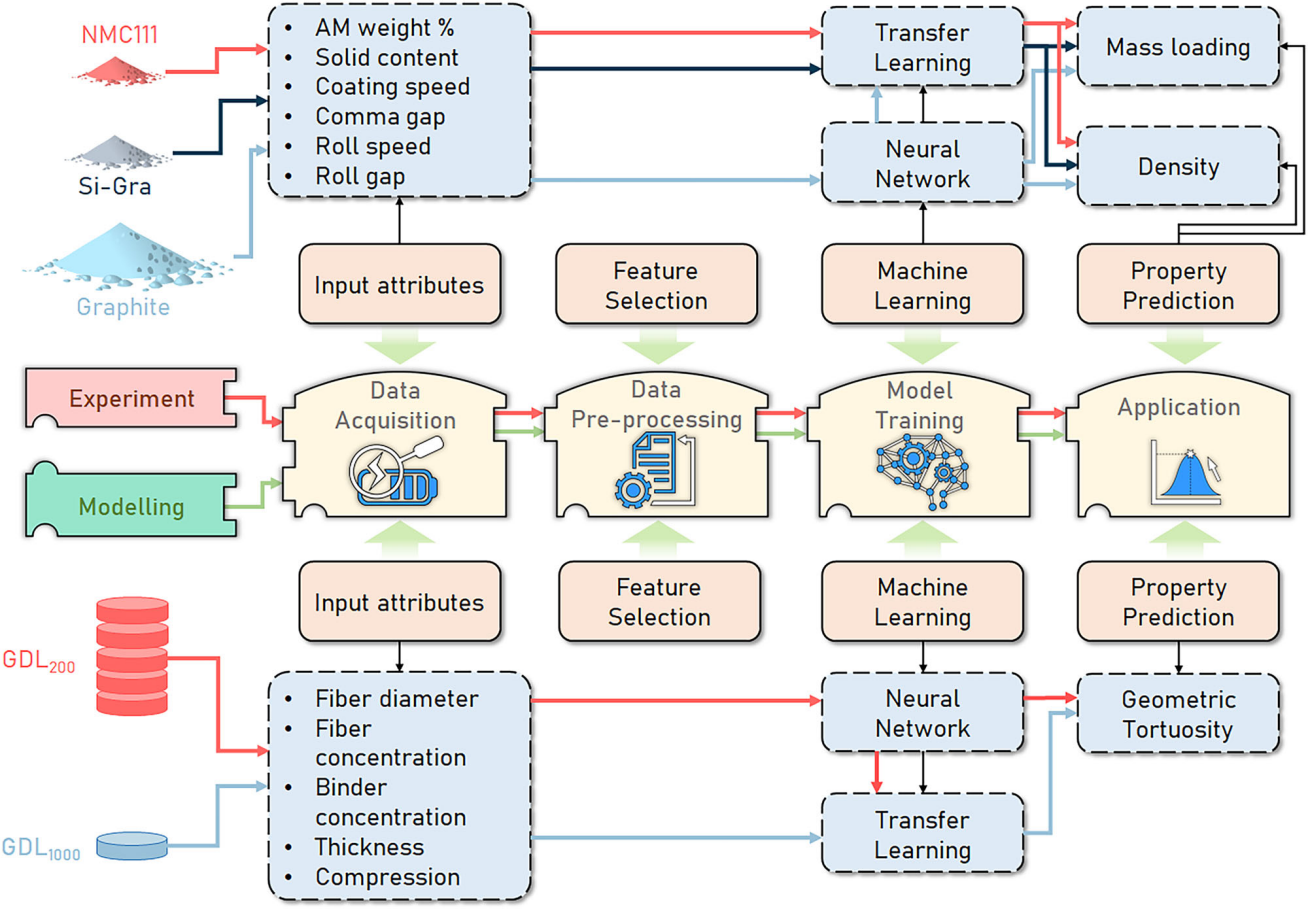
Our workflow for the application of the simple Transfer Learning (TL) approach. In the upper part of the diagram, we have the case of applying it to an experimental dataset to predict electrode density and mass loading using the Graphite dataset as the vast one and transferring it through the TL approach to the Silicon-Graphite (Si-Gra) and the NMC smaller datasets. In the lower part of the diagram, we have the case of applying the TL approach to the stochastically generated dataset (GDL_200_ as a source dataset and GDL_1000_ as a target dataset, explained in the Methods section) to predict the GDL geometric tortuosity.

In this present work, we propose a simple TL approach to understand how relationships between manufacturing parameters and components properties can be transferred to different chemistries with experimental data, and to different volume sizes with stochastically generated data (Fig. 1). This proposed TL approach allows dealing with small datasets, which may be the case in academic research laboratories. In the experimental demonstration, we used a larger Graphite dataset for preparing LIB negative electrodes to train an NN and transfer it to Silicon-Graphite (Si-Gra) and NMC smaller datasets for preparing negative and positive electrodes respectively. We consider the AM weight percentage and the solid content in the electrode slurry formulation, the coating speed and the comma gap in the coating step, and the roll speed and the roll gap in the calendering step. These six manufacturing parameters are considered to predict the electrode density and mass loading. Meanwhile, for GDLs, we use a larger dataset with calculation results for small volume microstructures to train an NN and transfer it to a smaller dataset with fewer calculations but with larger volume microstructures that are computationally more expensive to obtain. In this case, we predict the geometric tortuosity by considering as input parameters the fiber diameter, the fiber concentration, the binder concentration, the thickness, and the compression factor. It can be highlighted that none of these datasets were specifically generated for the purpose of training a ML model. Therefore, their distributions were not specifically designed for this application, but our approaches here allow us to obtain good results despite this aspect. In the first case, the dataset is heterogeneously distributed and in the second one it is uniformly distributed, which shows the robustness of the method when applied to different distribution types. In the following Section, we explain our findings from applying these approaches to our manufacturing process experimental dataset related to LIBs cells and stochastically generated dataset related to PEMFCs. We also use Explainable Artificial Intelligence (XAI) to interpret the behavior of our NN’s predictions before and after the TL approach is applied. Finally, we conclude with a discussion of the results obtained and indicate the future perspectives of our work.

## Results

### Experimental LIB cell manufacturing dataset

**Fig. 2:**
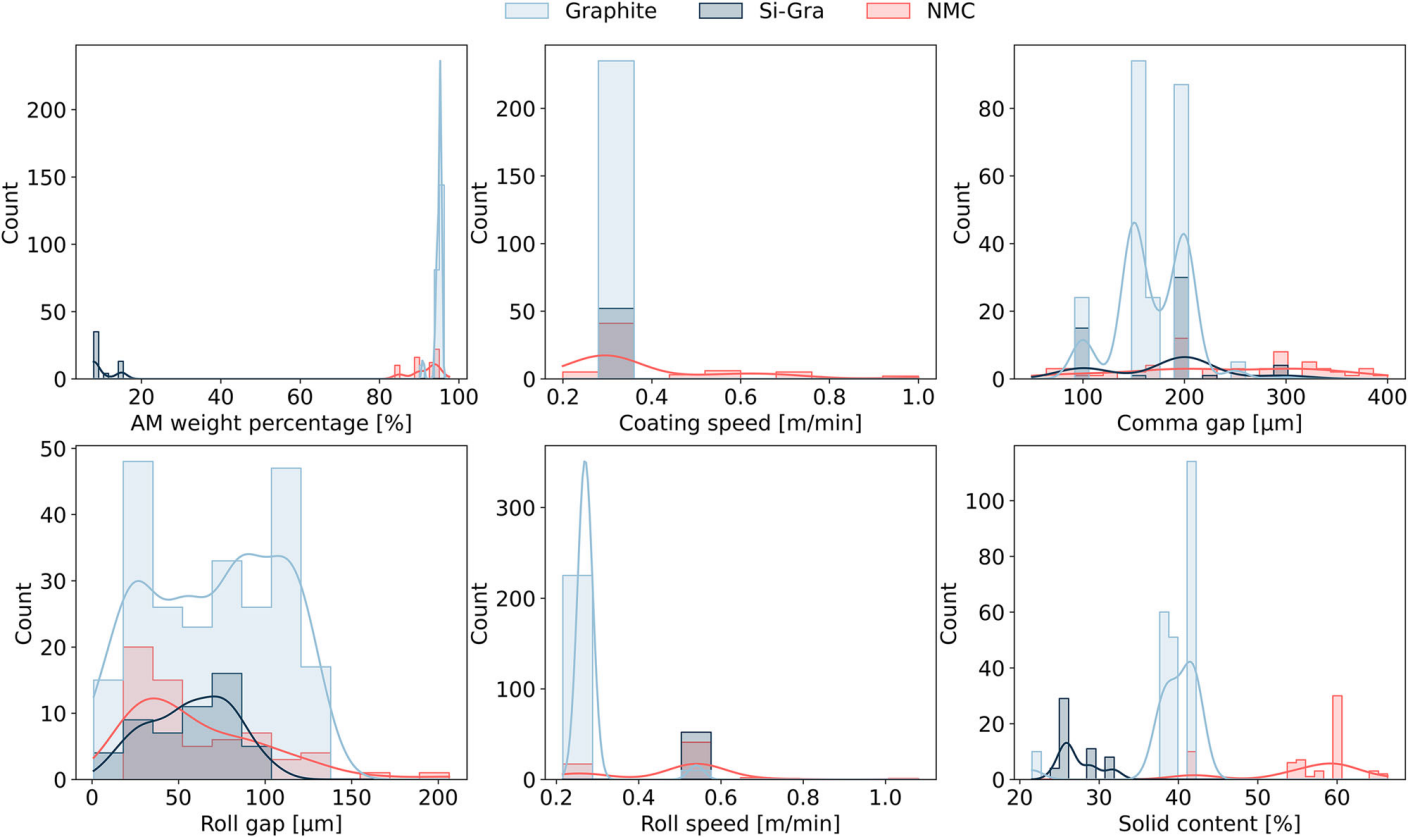
Experimental manufacturing parameters analysis. Distributions of the manufacturing parameter values in the pre-existing experimental dataset used as input features in the Neural Networks (NNs) for each Lithium Ion Battery electrode Active Material (AM): Graphite (light blue), Si-Gra (dark blue), and NMC (red).

Overall, our pre-existing experimental dataset consists of 235 Graphite-based, 53 Si-Gra-based, and 63 NMC-based electrodes that were fabricated under the conditions described in the Methods section. Figure 2 shows the distribution of the experimental manufacturing parameters for each AM. As global aspects of these distributions, we can highlight that Graphite is the most extensive one, in terms of the range of values considered and the amount of data that can be appreciated in the counts *y*-axis. Then, Si-Gra and NMC have parameter values in the same range except for some cases such as the AM weight percentage in Si-Gra or solid content in NMC. This makes the decision to use TL a good one, as the added extra layers (1 in our case) are expected to adapt the model to each of these particularities. The goal of this work is to learn the impact of these six input features (AM weight percentage and solid content in the formulation, coating speed and comma gap in the coating step, and roll speed and roll gap in the calendering step) on the final electrode properties (density and mass loading) from Graphite-based electrodes and then apply the TL approach to Si-Gra- and NMC-based electrodes. The size of these datasets reflects a typical experimental case where their acquisition requires a significant amount of resources. Such situations may recur in academic laboratories and therefore the TL approach proposed here aims to address them.

The AM weight percentage varies from 85% to 97.5% for NMC and from 91% to 95.5% for Graphite. For the particular case of Si-Gra, the AM weight percentage is considered to be equal to the weight percentage of Si in the Si-Gra composite electrode and ranges from 8% to 15% (even though, Graphite functions also as an AM in the operating electrode). A coating speed value of 0.3 m/min is considered for Graphite and Si-Gra, while for NMC more cases between 0.2 m/min and 1 m/min are considered. Regarding comma gaps, the range of values goes from 100 to 300 µm for Graphite and Si-Gra electrodes and from 50 to 400 µm for NMC electrodes. For the roll gap, we refer to the initial gap between the two calendering rolls before the electrode (supported on the current collector) passes between the rolls. During the calendering process, this gap can change to accommodate both the electrode and the current collector. The values of the roll gap have been previously calibrated to produce different pressures for different values of the calendering gap^42,64^. For example, Primo et al. from our group calibrated the dependence of the pressure on the roll gap for a given electrode^42^. While for Graphite the minimum and maximum values are 1 µm and 135 µm, the values for Si-Gra and NMC are 15 µm and 102 µm, and 20 µm and 206 µm, respectively. Their respective mean values are 70 µm, 58 µm and 62 µm. For the roll speed we have two possible values of 0.27 m/min and 0.54 m/min. Finally, the solid content varies from 42% to 66% for NMC and from 22% to 42% for Graphite slurries, since the Graphite slurry is more viscous at the same shear rate and formulation^65,66^ due to the choice of the binder, and the solid content for Si-Gra slurries are close to the lower values of Graphite (from 24% to 32%).

**Fig. 3:**
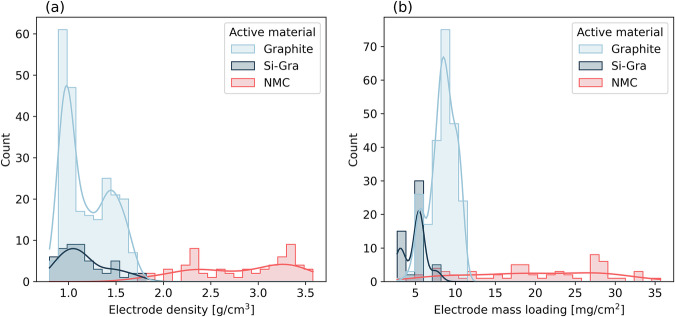
Experimental electrode properties analysis. Distributions of the target electrode properties for the NNs: **a** Density and **b** Mass loading. In each plot the electrode AMs are represented by the following colors: Graphite (light blue), Si-Gra (dark blue), and NMC (red).

The final electrode properties considered for prediction in this work are the density and the mass loading, whose distributions are shown in Fig. 3. The electrode density, along with the electrode mass loading, are important properties that control the final performance of the LIB cells. Increasing electrode mass loading and density generally increases energy density. However, this often results in a decrease in power density^67^. The optimization of these properties is always a balancing act and is also highly dependent on the type of electrode. As we can see in Fig. 3a, Graphite- and Si-Gra- based electrodes are generally less dense than NMC-based electrodes due to the lower mass density of Graphite and Si compared to NMC, resulting in a lower value of the maximum electrode density, leading to differences in the range of the possible values of each electrode. In Fig. 3b, the mass loading of NMC-based electrodes is broader and considers larger values than the other two AMs. The higher mass loading is due to two factors. NMC has a higher density than Graphite, resulting in a higher mass of AM in the same volume of the electrode. Also, because solid content of the NMC slurry phase is higher, a higher amount of NMC is on the same area of the current collector for the same comma gap compared to Graphite or Si-Gra, resulting in a higher mass loading of NMC electrodes compared to these other electrode AMs.

**Fig. 4:**
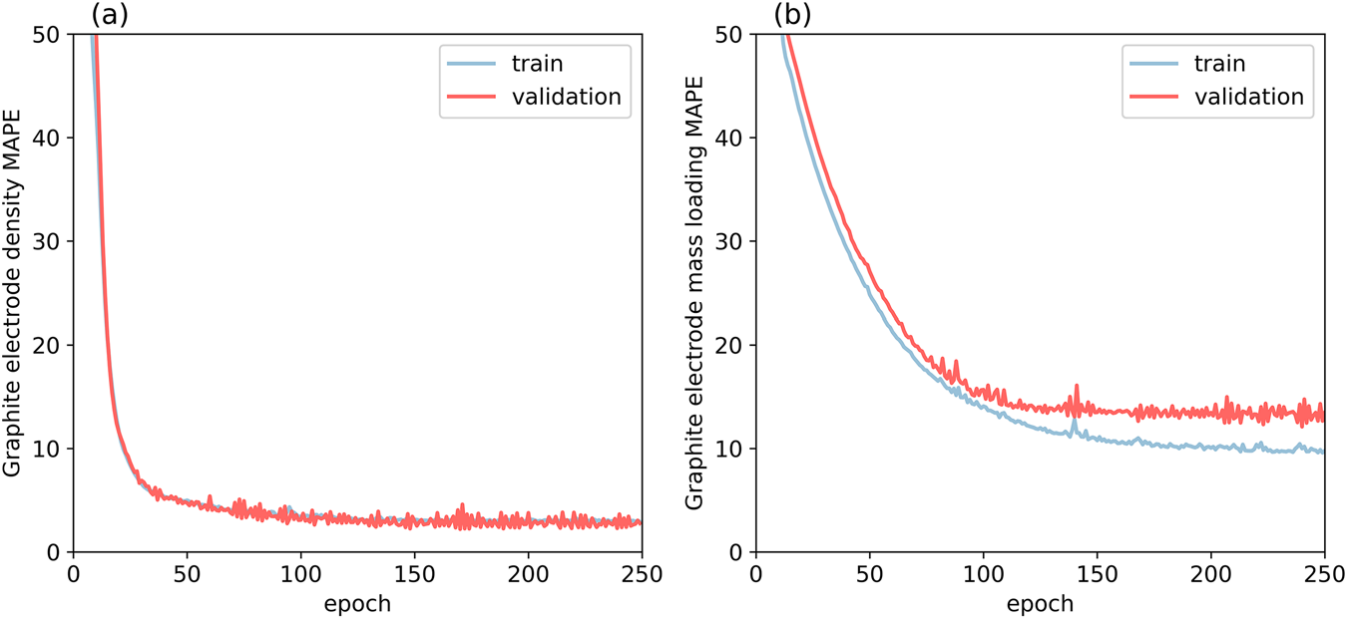
Training/validation loss curves. Mean Absolute Percentage Error (MAPE) loss plot for electrode (**a**) Density and (**b**) Mass loading for both training (blue curve) and validation (red curve) sets for the NN trained on the Graphite electrode dataset.

In this experimental demonstration of our simple TL approach, we studied the dependence of electrode density and mass loading on the fraction in mass of AM and percentage of solid content in the formulation, coating speed and comma gap during coating, and roll speed and roll gap during calendering. To simplify the subsequent application of TL techniques and training, we have split the problem into two prediction problems, i.e., prediction of the electrode density and prediction of the electrode mass loading. To demonstrate that the Graphite dataset is vast enough to be considered as the source dataset in the TL application, we have performed an ablation study, which is shown in Figure S2. It is worth noting that the error converges to a plateau before reaching the full size of the dataset, demonstrating that this Graphite dataset is vast enough for this TL application. A random train-test split of 70%-30% was performed on the dataset for each electrode. Then, 10% of the 70% training set was used as the validation set during the training process. The optimization loss function was the Mean Absolute Percentage Error (MAPE) and the Adam optimizer was used per 250 epochs. We have studied the sensitivity of the pre-trained NNs with the percentage considered in the training-testing split in Figure S1 of the Supporting Information. We chose this split over the most common 80–20% split because of the amount of data that we have in the smaller datasets (given that they have similar errors). For example, we have 53 Si-Gra electrodes, which with the 80%-20% train/test leaves only 11 electrodes for testing. Meanwhile, with the chosen split percentages, we have 16 electrodes for testing. Figure 4 shows the MAPE loss of the NN with the Graphite dataset for both density and mass loading cases. For both models, the loss decreases with an asymptotic shape until it reaches a plateau for both the train and the validation sets. This indicates that optimal weights of the NNs are found and there is neither over- nor under-fitting. The following results in plots and tables correspond to the evaluations on the test set.

Having obtained the good performance of the pre-trained NNs for Graphite electrode dataset, which could capture the relationships between the experimental manufacturing parameters and electrode properties, we now extend them to Si-Gra and NMC smaller datasets. Training such models for these two electrode datasets with the lack of data leads to overfitting in the respective training set, as shown in Fig. S3 and S4 of the Supporting Information. Therefore, the goal here is to adapt these pre-trained NNs to the new chemistries using the proposed TL approach. To overcome this problem, we freeze the weights in the connections between the layers of the pre-trained NN architecture for the Graphite electrode dataset and add an extra hidden layer to each of the other chemistries NNs, with the same number of nodes of the corresponding pre-trained NN. Thus, since only the weights of this layer are trained, the problem adapts better to the amount of data of Si-Gra and NMC datasets. The train and validation losses for the training of the TL-based NNs in these smaller datasets are shown in Fig. S5 and S6. The need for these additional layers can be justified with the results shown in Table 1. The pre-trained NN for predicting electrode density gives an error of 4.5% when evaluated with Graphite-based test data. The error of this model increases up to 78.8% and 56.1% when tested on Si-Gra and NMC test data, respectively. This is because the pre-trained NN extrapolates outside the feature range for which it was trained, as seen in Figs. 2 and 3. In the next row of Table 1, we can see a decrease in the Si-Gra and NMC test errors, where these errors decrease to 14.5% and 8.1%, respectively, demonstrating the improvement provided by the TL-based NN when the additional layer is added. For the pre-trained NN that predicts the electrode mass loading, we have a similar behavior where the error is 13.9% when evaluated in Graphite test data and increases to 45.0% and 70.8% for the Si-Gra and NMC test data, respectively. These errors decrease to 3.7% and 10.6% when the simple TL approach is applied. These errors can also be compared to the errors we get when we train different baseline models on our pre-existing experimental datasets (Table S1 in the Supporting Information). These are an average prediction (a model that predicts the average of the target property in the training set), a linear regression, and an RF model. The errors of some of these baseline models are of the same order as those of the NNs. In other cases, however, the errors of the NNs (both pre-trained and TL-based) are significantly smaller. Furthermore, in contrast to these baseline models, the TL approach has the advantage of allowing us to adapt a pre-existing model (here, the pre-trained NN in the Graphite dataset) and then train it on smaller datasets (the TL-based NN in the Si-Gra and NMC datasets).

**Table 1:** Mean Absolute Percentage Error (MAPE) of density and mass loading predictions in the test sets for both pre-trained and TL-based NNs

	Error (MAPE) [%]					
	Density			Mass loading		
	Graphite	Si-Gra	NMC	Graphite	Si-Gra	NMC
Pre-trained NN	4.5	78.8	56.1	13.9	45.0	70.8
TL-based NN	--	14.5	8.1	--	3.7	10.6

The standard deviation of the reported errors is 0.5% and was estimated by performing a K-fold cross-validation with 5 random train-test splits. The specific error of each one of the folds is given in Table S2 of the Supporting Information.

**Fig. 5:**
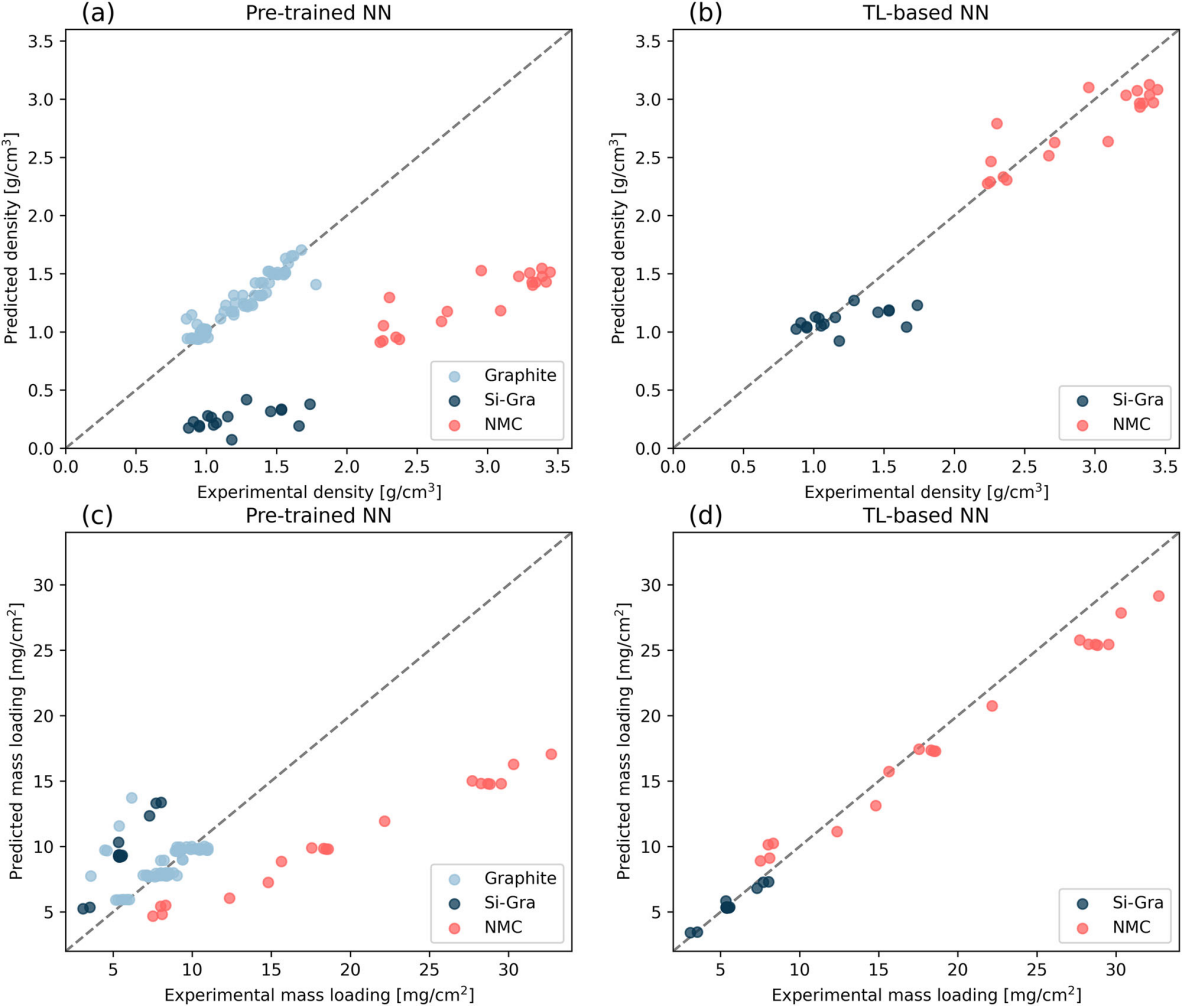
Scatter plot of the predictions versus targets. Predicted versus experimental values of: **a** Density (pre-trained NN), **b** Density (TL-based NN), **c** Mass loading (pre-trained NN) and **d** Mass loading (TL-based NN) in the test sets. In each plot, the electrode AMs are represented by the following colors: Graphite (light blue), Si-Gra (dark blue), and NMC (red).

Figure 5 shows this in a more graphical way, where each prediction is plotted against its actual value. In the left column of the plots, we have the predictions from the pre-trained NNs. Based on the MAPE results presented in Table 1, good performance is not expected for the Si-Gra and NMC test data. In the right column, we have the TL-based NNs, so an improvement is expected with respect to the left column. Figure 5 show the linear trend of an ideal model as a dashed gray line in each subfigure. For both pre-trained NNs (Fig. 5a and c), the predictions of Graphite test data are shown, where in the case of electrode density we obtain a good behavior, while in the case of electrode mass loading an artifact of overestimation of low mass loading values is generated. For both Si-Gra and NMC electrodes, the actual density is underestimated and the Si-Gra mass loading is overestimated. Figures 5b and d show how the TL-based NNs correct this, with Si-Gra and NMC predictions improving within the range of expected values. However, the relative differences between them remain unchanged in the electrode density predictions. Futhermore, predictions are improved for the mass loading, where even the slope of the trend is improved for both AMs. Thus, the simple TL approach allowed us to generate new models with great accuracy despite the data size limitation.

**Fig. 6:**
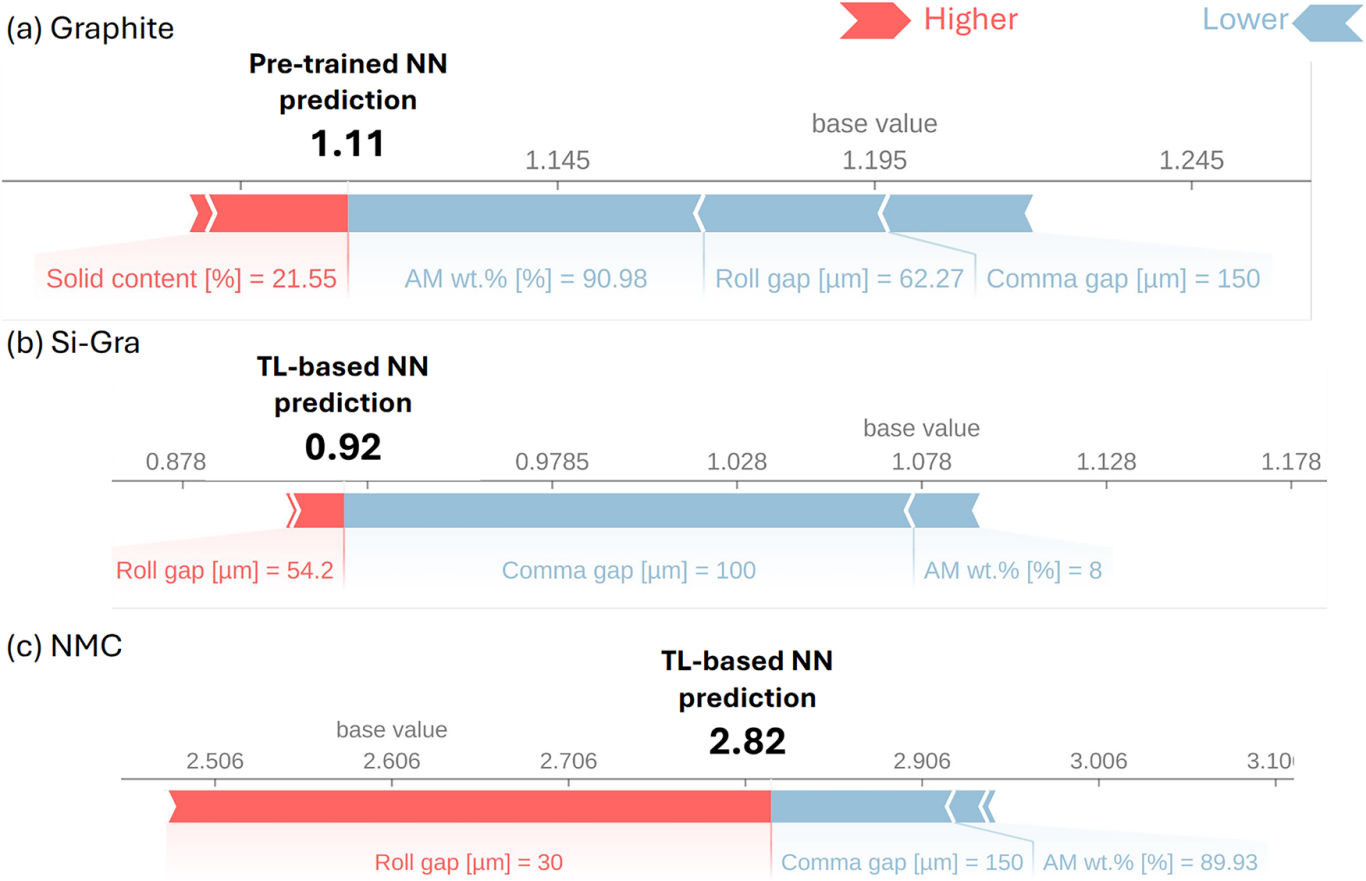
SHAP values for single prediction explanation. Representation of SHAP values for the NNs prediction of the electrode density for a randomly selected test experiment from **a** Graphite, **b** Si-Gra, and **c** NMC electrode datasets. These plots show how the input features of a given data point contribute to the prediction of the NN by showing the magnitude and direction of the contribution as arrows with different colors for increase (red) or decrease (blue).

To add explanation to the predictions of the NNs, we use XAI, which is a technique used in AI to add interpretability and transparency to the usual black-box ML models. In Fig. 6 we show the SHapley Additive exPlanations (SHAP) values for the pre-trained and the TL-based NNs for a randomly selected test experiment for each chemistry. These SHAP values compute the importance of each feature close to a fixed data point when all the other feature values are constant^68^. This allows us to compare the impact of each manufacturing parameter on the final electrode property for the different chemistries when evaluating a given experiment. In Fig. 6, the base value indicates the average of all predictions in the test set of the given chemistry. The red or blue color of the arrows in these plots indicates whether the associated manufacturing parameter, with the specified value and unit, is pushing the electrode density prediction to a higher or lower value relative to that base value. The magnitude of each feature’s contribution is indicated by the size of the corresponding arrow. When all of these arrow lengths are added to the base value, with the sign indicated by the color, the final prediction is obtained. For the experiment selected from the Graphite test set, we have a strong influence of the AM wt.%, roll gap and the comma gap by pushing the electrode density prediction to the negative side of the base value, while the solid content pushes it to the positive side. While for the selected experiments of Si-Gra and NMC respective test sets, the influence in their electrode density prediction is dominated in both cases by the roll gap, the comma gap, and the AM wt.%, with the same direction on each one but with different magnitudes. This XAI allows the further improvement of these specific experiments by indicating which manufacturing parameters have more influence on the electrode property.

**Fig. 7:**
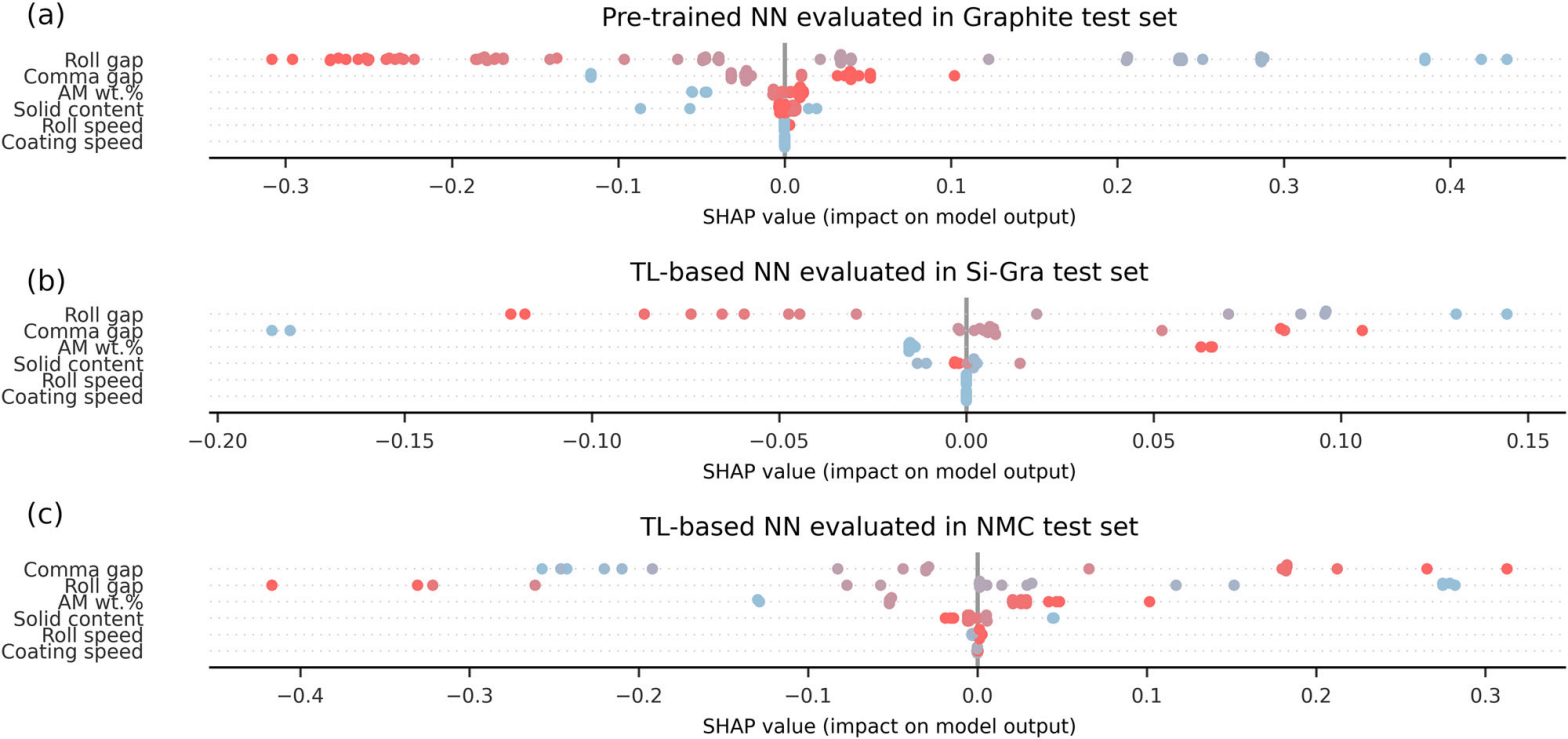
Contribution of each manufacturing parameter to the predictions. Global representation of SHAP values for the NNs predictions of the electrode density for each experiment in **a** Graphite, **b** Si-Gra, and **c** NMC electrode test sets. The SHAP value of each manufacturing parameter for each test experiment is given on the *x*-axis, while the color of each point indicates whether the feature value is higher (red) or lower (blue) relative to its distribution. The *y-*axis is ordered by the relevance manufacturing parameters to the NN prediction, with the most relevant at the top.

To have a global overview of the importance of each manufacturing parameter in the prediction of the electrode density, we summarize in Fig. 7 the evaluation of the SHAP values for each experiment in the test sets. Here each point represents a specific experiment in the test set. For Graphite experiments, the pre-trained NN was used, while for Si-Gra and NMC, the respective TL-based NNs were used. The color of each dot indicates the value of the feature (relative to its distribution), while the *x*-axis positions indicate the individual influence in the NN prediction (SHAP value). The *y*-axis of each subfigure is sorted in descending order of importance of each manufacturing parameter. We can highlight that the impact of the manufacturing parameters on the NN prediction depends on the chemistry. This peculiarity indicates that the TL approach can modify the relevance of the manufacturing parameters in the pre-trained NN when transferred to a new dataset. While for Graphite and Si-Gra pre-trained and TL-based NNs, respectively, the manufacturing parameters that have a greater influence on the outputs are roll gap, comma gap, AM wt.% and solid content. For the NMC TL-based NN, the three most important manufacturing parameters change their order with respect to the previous ones: comma gap, roll gap, and AM wt.%. It can be highlighted that in all cases, the roll speed and the coating speed have no influence on the NN’s predictions. This can be attributed to their distributions (see Fig. 2), which are composed of some specific discrete values. This should not be interpreted as implying that these manufacturing parameters do not have influence on the final electrode properties, but rather as a characteristic of the trained NNs, influenced by the particularities of the pre-existing experimental dataset. Moreover, this global representation could be used to select features and consider a less complex model without taking into account these manufacturing parameters for this case or to improve their experimental sampling.

### Stochastically generated GDL manufacturing dataset

The overall performance of the proposed TL approach might not be perfect, considering the percentage errors for some cases presented in Table 1. However, this could be attributed to the peculiar distribution of the pre-existing experimental dataset. This is where our stochastically generated GDL manufacturing dataset comes in. The source GDL_200_ dataset has 240 entries, while the target GDL_1000_ dataset has 78 entries. These sizes maintain a similar ratio between the source and target dataset sizes of the experimental demonstration. However, because they are stochastically generated, they have a more homogeneous distribution, providing further evidence of the validity of this TL approach. The distributions of the input features for the NN to predict the geometric tortuosity are shown in Figure S7. The GDL_200_ dataset shows a uniform distribution. While the GDL_1000_ dataset shows some peculiarities due to the smaller amount of data. For both datasets, we have similar ranges for each input parameter as these were the ranges defined for generating the GDL microstructures. Fiber diameters range from 6.0 to 12.0 µm. Fiber and binder concentrations vary from 10% to 25% and from 5% to 10%, respectively. The thickness ranges from 280 to 320 µm, and finally the compression factor ranges from 0.0 to 0.5. Here we define the compression factor as the percentage of compression applied along the *z*-axis of the GDL microstructure. More details about these considerations can be found in our previous work^35^.

**Fig. 8:**
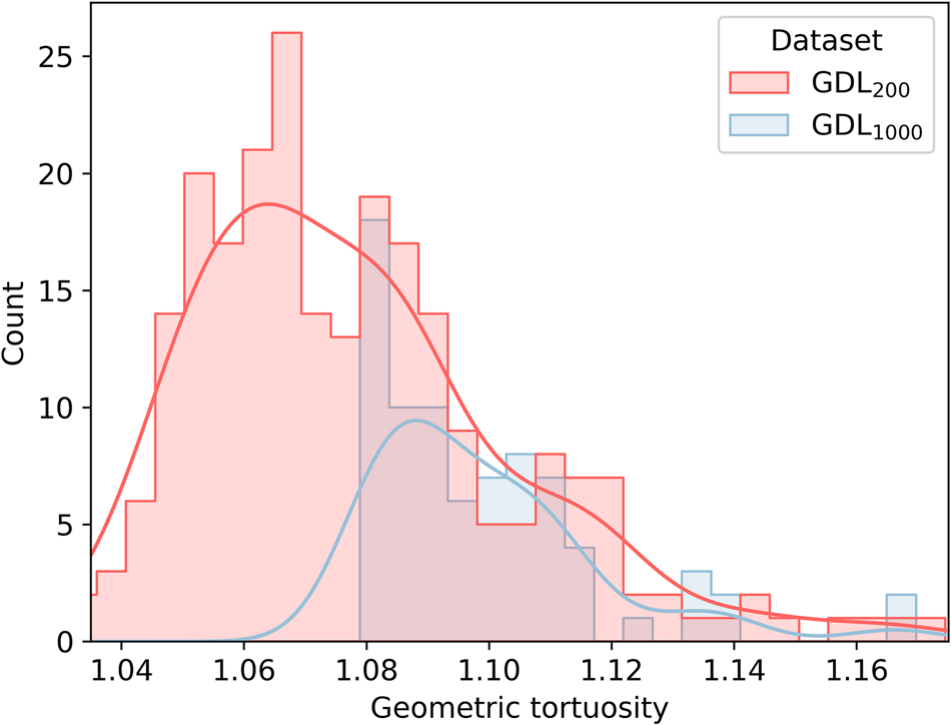
Geometric tortuosity characterization of the stochastically generated datasets. Distributions of the target GDL geometric tortuosity for the NNs for each dataset: GDL_200_ (red) and GDL_1000_ (blue).

The distributions in each dataset of the geometric tortuosity are shown in Fig. 8, which is the target to be predicted by the NN in this subsection. The geometric tortuosity of the GDL is an important property because it plays a critical role in the transport of gases, liquids, and heat within the system. It also affects the permeability, which ultimately affects the efficiency and performance of the PEMFC. Higher geometric tortuosity means longer diffusion paths, which affects the transport properties of the GDL. It can be noted that both datasets (the GDL_200_ and the GDL_1000_) the geometric tortuosity distributions are right-skewed, and the GDL_1000_ geometric tortuosity values are shifted to the right of the GDL_200_ ones. While the mean value for the GDL_200_ is 1.07, the mean value for the GDL_1000_ is 1.09. Also, the minimum value in the GDL_200_ is 1.03, while in the GDL_1000_ is 1.08. Both of the distributions go up to 1.17.

For the training of the NNs, a random train-test split of 70%-30% was performed on each dataset, with the first one being used only for training and the second one for the test evaluations performed in the following tables and plots. As in the previous case, the optimization loss function was the MAPE, and the Adam optimizer was used per 250 epochs. Figure S8 shows the MAPE loss of the NN trained on the GDL_200_ dataset, where the loss decreases with an asymptotic behavior and reaches a plateau close to 2%. When this pre-trained NN is evaluated in the test sets, we have a MAPE value of 2.1% for the GDL_200_ and of 2.6% for the GDL_1000_. This higher error for the GDL_1000_ can be improved by applying the TL approach, which has already shown improvements with the experimental dataset. This decision is also supported by Figure S9 in the Supporting Information, where we show the overfitting obtained when the NN is trained on the smaller GDL_1000_ dataset. This justifies the use of the TL approach also on this stochastically generated dataset. By adding and training the extra layer to the pre-trained NN, the evaluation on the GDL_1000_ test set is reduced to 2.0%. The train and validation losses for the training of this TL-based NN on the smaller GDL_1000_ dataset are shown in Fig. S10. This information is summarized in Table 2, which shows that there is also an improvement with the TL approach when applied to this stochastically generated dataset.

**Table 2:** Mean Absolute Percentage Error (MAPE) of geometric tortuosity predictions in the test sets for both pre-trained model and TL-based NNs

	Geometric Tortuosity MAPE [%]	
	GDL_200_	GDL_1000_
Pre-trained NN	2.1	2.6
TL-based NN	--	2.0

The standard deviation of the reported errors is 0.2% and was estimated by performing a K-fold cross-validation with 5 random train-test splits. The specific error of each one of the folds is given in Table S2 of the Supporting Information.

**Fig. 9:**
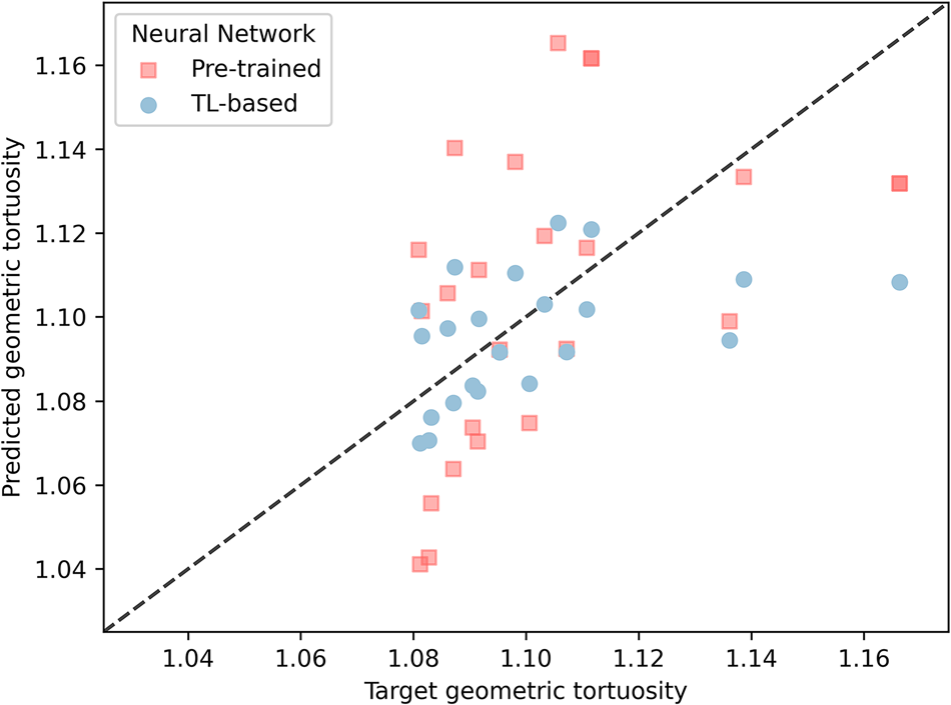
Scatter plot of predictions versus targets. Predicted versus target values of Geometric Tortuosity in the test set of the GDL_1000_ test set with the pre-trained NN (red squares) and with the TL-based (blue circles) NNs.

The individual predictions are plotted against the target values for the GDL_1000_ test set and both pre-trained (red squares) and TL-based (blue circles) NNs in Fig. 9. As expected from the values in Table 2, the predictions of the pre-trained NN have a higher scatter on the predicted *y*-axis. This scatter is reduced with the TL-based NN by bringing each prediction closer to the ideal prediction line. This improvement is clearer in the range that goes from 1.08 to 1.12. Then, the three cases with higher geometric tortuosity are worse. However, these values are in the tail of the distribution and are less sampled than those in the improved range.

Finally, we also use XAI in these datasets where entries of the GDL_200_ and GDL_1000_ test sets are randomly selected and evaluated with the pre-trained and the TL-based NNs, respectively. The obtained SHAP values are shown in Fig. S11. In the first case, we have a large influence in the negative direction with respect to the base value, which is dictated by the thickness, the fiber concentration, and the fiber diameter. While the compression factor and the binder concentration have similar contributions but in the opposite direction. For the second case, the compression factor is the only parameter that pushes the prediction in the negative direction. While all the other parameters push it to a higher value, the thickness and the fiber concentration are the most relevant ones for this test example. Going further, we can repeat this analysis for all the samples in the respective test sets and evaluate them with the respective NNs. When this information is plotted together (Fig. S12), we get an overview of the influence of each feature in the NNs predictions. In these stochastically generated GDL datasets, we see that the influence of the features in both the pre-trained and the TL-based NN are in the same order of importance, i.e. there is no significant change in the relative importance of each feature to the others when making predictions. As it can be seen in Fig. S12, the order of the importance of the features is as follows: thickness, fiber concentration, binder concentration, fiber diameter, and compression factor. Thickness is the most relevant one and compression factor is the least relevant one in determining the geometric tortuosity of the GDL microstructure datasets with our pre-trained and TL-based NNs.

## Discussion

The novel and simple TL approach proposed in this work has been shown to be suitable for EEC manufacturing problems. It has been demonstrated on pre-existing experimental and stochastically acquired datasets, which were not specifically designed to demonstrate our TL approach. The transferability of the methodology to different systems in the context of EEC manufacturing was proved by considering LIB electrodes and PEMFC GDLs datasets. These datasets came from different sources: in the case of LIB electrodes from experiments and in the case of GDLs from (computational) stochastic generations. In addition to all these aspects, the datasets considered presented different distributions, a heterogeneous one for LIB electrodes and a uniform one for PEMFC GDLs. Given all these considerations and the excellent performance obtained for both cases, we have demonstrated the robustness of our TL approach to address EEC manufacturing problems with small datasets.

In the first application, the experimental dataset consisted of manufacturing process data of LIB electrodes with different AM chemistries. The approach consisted of using a vast Graphite dataset to train NNs to predict electrode density and mass loading. These NNs were trained using the following manufacturing parameters as features: weight percentage of AM and solid content in the slurry formulation, slurry coating speed and comma gap during coating, and roll speed and roll gap during electrode calendering. The architecture of the NNs was designed differently for each target property. These NNs performed well on the Graphite electrode dataset, but their performance metrics diminished when used to extrapolate in the smaller Si-Gra and NMC electrode datasets. The distribution of these Si-Gra and NMC datasets had both similarities and differences with the Graphite dataset, but they were not large enough to train NNs from scratch. This justified the use of TL by adding an extra layer to each one of the Graphite pre-trained NNs and training this extra layer to adapt these models to each one of these new chemistries. In both cases, the performance of the evaluation metrics improved significantly when evaluated with the TL-based NN predictions. The interpretation of the influence of each parameter in the NNs predictions was also discussed for some test experiments for each chemistry using XAI. The global analysis of SHAP values allowed us to determine the influence of each manufacturing parameters on the final predicted electrode properties and to sort them by relevance, showing that this depends on the type of chemistry.

The second application of the simple TL approach was in the context of PEMFCs with stochastically generated GDL datasets with different volume sizes. In this case, small-volume microstructures characterized with numerous calculations were used to train a NN to predict the geometric tortuosity, considering as input parameters the fiber diameter, the fiber concentration, the binder concentration, the thickness, and the compression factor. The NN was then transferred to a smaller dataset of larger volume microstructures that were computationally more expensive to characterize. Since these datasets were stochastically generated, they have a more homogeneous distribution than the experimental ones. This property of the data led to even better results, proving that the simple TL approach can perform better when applied to better distributed datasets.

This work provides a proof-of-concept for developing efficient data-driven models with the simple TL approach to predict final electrode properties for new chemistries or large simulation volume sizes by taking advantage of pre-trained NNs when available data is not enough (as it may occur in academic laboratories). Our work perspectives include the application of our approach to LIB electrode formulations with other chemistries and to sodium-ion and Solid-State Battery electrode manufacturing processes. We believe that the proof-of-concept presented in this article illustrates that simple AI approaches can still deliver a lot in the complex field of EEC manufacturing.

## Methods

### Sample preparation and properties measurement in LIBs

Our pre-existing experimental dataset consisted of three different chemistries for the LIB electrode AM: Graphite, Silicon-Graphite composite (referred to as Si-Gra), and NMC. It is worth mentioning that it was not acquired with the purpose of demonstrating the TL approach proposed in this work. For the positive electrode, NMC AM, supplied by Umicore, was used. C-NERGY™ super C45 carbon black (CB) from IMERYS and Solef™ Polyvinylidene fluoride (PVDF) from Solvay were used as the electronic conductive additive and the binder, respectively. Prior to mixing with the solvent, the powder components were first premixed overnight in a Turbula^®^ mixer. The mixture was then transferred to a Dispermat CV3-PLUS high-shear mixer, and the required amount of NMP (BASF) was added. The resulting mixture was mixed for 2 hours at 25 ^o^C and 3000 RPM. For the graphite electrode, Na-CMC (molecular weight ∼250 K and degree of substitution ∼0.7, Sigma Aldrich), C-NERGY™ super C45 CB (IMERYS), and water were used as the binder, conductive additive, and solvent, respectively. The slurry mixing step was similar to the NMC slurry. For some of these graphite electrodes, Si nanoparticles were also used as AM mixed with Graphite to fabricate Si-Gra composite electrodes. The total AM content is fixed at 91%, and the total weight percent of Si is varied to produce different formulations of the electrodes.

The resulting slurries were coated over a copper current collector (16 μm) for Graphite and Si-Gra electrodes and an aluminum current collector (22 μm) for NMC electrodes with a prototype-grade comma-coater machine (PDL250, People & Technology, Korea) at various comma gaps and coating speeds as discussed in the experimental case of the Results. The electrodes were dried in a built-in two-part oven at 80 and 95 °C for NMC electrodes and at 60 and 65 ^o^C for Graphite and Si-Gra electrodes due to the different solvents used. Calendering of the electrodes was performed on a prototype-grade lap press calender (BPN250, People & Technology, Korea) at various roll gaps and roll speeds. The temperature of the calendering process was maintained at 60 ^o^C for all the electrodes.

The resulting electrodes were characterized in terms of density and mass loading, which are two final structural properties of the electrode which can greatly influence its achievable electrochemical capacity. A sample of 13 mm diameter electrode sections was punched from the electrode. The thickness and mass of the resulting electrode section were measured, and the thickness and mass of the current collector were subtracted to obtain the final mass and thickness of the electrode. Mass loading (${m}_{l}$) of the electrode was calculated as

1 $${m}_{l}\left(\frac{{mg}}{{{cm}}^{2}}\right)=\frac{{f}_{{AM}}\,\times \,({m}_{{el}+{cc}}-{m}_{{cc}})\times \,4}{\pi \times \,{1.3}^{2}}$$

where, ${m}_{{el}+{cc}}$ is the mass of the electrode along with the current collector, ${m}_{{cc}}$ is the mass of the current collector, and ${f}_{{AM}}$ is the total fraction of the active materials. The density ($\rho$) of the final electrode was calculated as:

2 $$\rho \left(\frac{g}{{{cm}}^{3}}\right)=\frac{{m}_{l}\times \,0.001}{{f}_{{AM}}\times \,({t}_{{el}+{cc}}-{t}_{{cc}})}$$

where ${t}_{{el}+{cc}}$ is the thickness of the electrode along with the current collector, in cm, and ${t}_{{cc}}$ is the thickness of the current collector, in cm.

The slurry formulation, the coating and drying process parameters, and the calendering process parameters are used as the input features of the model. The slurry is mixed at the same mixing speed and for a sufficient time for each type of electrode to ensure a homogeneous mixture. For the formulation, the solid content of the slurry and the weight percentage of AM are varied. The same weight percentage of carbon additive and binder is taken for all the formulations. The solid content of the slurry is defined as,

3 $${\rm{Solid}}\; {\rm{content}}\,\left( \% \right)=\frac{{\rm{dry}}\; {\rm{mass}}\,({\rm{i}}.{\rm{e}}.{\rm{mass}}\; {\rm{of}}\; {\rm{AM}}+{\rm{carbon}}\; {\rm{additve}}+{\rm{binder}})}{{\rm{dry}}\; {\rm{mass}}+{\rm{mass}}\; {\rm{of}}\; {\rm{solvent}}}$$

The coating and drying processes are performed roll-to-roll. The parameters controlling the coating and drying process are coating gap, coating speed, and drying temperature. Among these parameters, the drying temperature is fixed for each type of electrode according to the solvent, and the other parameters are varied. Finally, the dried electrodes are subjected to the calendering process, which is controlled by the roll speed, the roll gap, and the roll temperature. In our experiments, the roll temperature is fixed, and the roll gap and roll speed are varied. The fixed parameters for the mixing process were chosen given previous optimizations already performed in previous publications of our research group^42,66^. The experimental dataset used in the current work was obtained and collected in the context of the ARTISTIC project^30^.

### Stochastic Generation of GDL microstructures and calculation of geometric tortuosity

The GDL microstructures were generated using the FiberGeo module within the Geodict^69^ software. The microstructures were stochastically generated and digitally characterized according to several properties in the context of our previous work^35^. In that work, the geometric tortuosity was not used to train any ML model. The microstructures there consisted of infinite/circular carbon fibers with the specified solid volume percentage (SVP). We used them here with domain sizes of 2000 μm × 200 μm × thickness μm and 1000 μm × 1000 μm × thickness μm with a voxel length of 1 μm. These domain size datasets are referred to as GDL_200_ and GDL_1000_, respectively. In this case, the geometric tortuosity is our target property, whose digital characterization (determination of properties) in the GDL_200_ dataset requires 65% less computational time than in the GDL_1000_ dataset. The input parameters to calculate this target are the fiber diameter, the fiber concentration, the binder concentration, the thickness, and the compression factor.

The mentioned carbon fibers have a specified diameter and an isotropic orientation using an orientation tensor that can be explained as follows. Mathematically, $${{\bf{d}}}_{{\bf{k}}}=\left(\begin{array}{c}{{\rm{x}}}_{{\rm{k}}}\\ {{\rm{y}}}_{{\rm{k}}}\\ {{\rm{z}}}_{{\rm{k}}}\end{array}\right)$$ is the unit vector describing the **K**^**th**^ fiber and **n** is the number of fibers. The orientation tensor can be expressed as the sum of the dyadic products of **d**_**k**_ from all n fibers, divided by n:

4 $$\begin{array}{l}{\bf{T}}=\frac{1}{{\rm{n}}}\left(\mathop{\sum }\limits_{{\rm{k}}=1}^{{\rm{n}}}{{\bf{d}}}_{{\bf{k}}}{{\bf{d}}}_{{\bf{k}}}^{{\rm{T}}}\right)=\frac{1}{{\rm{n}}}\mathop{\sum }\limits_{{\rm{k}}=1}^{{\rm{n}}}\left(\begin{array}{ccc}{{\rm{x}}}_{{\rm{k}}}{{\rm{x}}}_{{\rm{k}}} & {{\rm{x}}}_{{\rm{k}}}{{\rm{y}}}_{{\rm{k}}} & {{\rm{x}}}_{{\rm{k}}}{{\rm{z}}}_{{\rm{k}}}\\ {{\rm{y}}}_{{\rm{k}}}{{\rm{x}}}_{{\rm{k}}} & {{\rm{y}}}_{{\rm{k}}}{{\rm{y}}}_{{\rm{k}}} & {{\rm{y}}}_{{\rm{k}}}{{\rm{z}}}_{{\rm{k}}}\\ {{\rm{z}}}_{{\rm{k}}}{{\rm{x}}}_{{\rm{k}}} & {{\rm{z}}}_{{\rm{k}}}{{\rm{y}}}_{{\rm{k}}} & {{\rm{z}}}_{{\rm{k}}}{{\rm{z}}}_{{\rm{k}}}\end{array}\right)=\left(\begin{array}{ccc}{{\rm{t}}}_{11} & {{\rm{t}}}_{12} & {{\rm{t}}}_{13}\\ {{\rm{t}}}_{21} & {{\rm{t}}}_{22} & {{\rm{t}}}_{23}\\ {{\rm{t}}}_{31} & {{\rm{t}}}_{32} & {{\rm{t}}}_{33}\end{array}\right)\\\qquad\qquad\qquad\qquad\;\;=\left(\begin{array}{ccc}0.4966 & 0 & 0\\ - & 0.4966 & 0\\ - & - & 0.0068\end{array}\right)\end{array}$$

where the diagonal elements define the orientation strength for the corresponding directions. After the generation of carbon fibers, the PTFE was incorporated into the GDL domain with the desired SVP. The geometric tortuosity ($\tau$) was then calculated to characterize the GDL microstructure. The calculation of the geometric tortuosity was conducted in the direction perpendicular to the direction of the carbon fibers using the following equation:

5 $${\rm{\tau }}=\frac{{\rm{shortest\; path\; to\; inflow\; plane}}}{{\rm{distance\; to\; inflow\; plane}}}$$

### Transfer learning approach

In our approaches herein, we use supervised ML by training feed-forward NNs on the pre-existing data collected from the LIB electrode manufacturing experiments and the data stochastically generated for GDL, as detailed in the previous respective subsections. NNs are chosen for their capacity to handle complexity. NNs can learn the underlying nonlinear relationships between the manufacturing parameters and the target properties. However, they also present some limitations: their complex structure can make them prone to overfitting if not carefully regularized and training them often requires large datasets and significant computational resources. To learn the weights of the NNs during training, the backpropagation algorithm is used^70^.

**Fig. 10:**
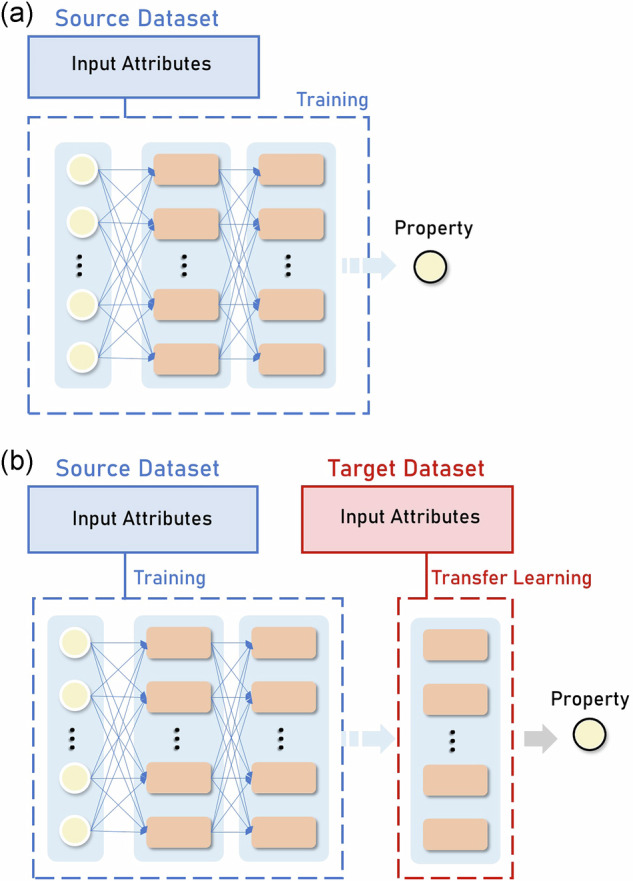
Explanation of pre-trained and TL-based NNs. Training scheme of a NN on (**a**) source dataset (Graphite-based electrodes for the experimental LIB electrode manufacturing dataset, and GDL_200_ for the stochastically generated GDL dataset), and then used for a (**b**) TL approach adding an extra layer for the target dataset (Si-Gra and NMC electrode datasets for the experimental LIB electrode manufacturing dataset, and GDL_1000_ for the stochastically generated GDL dataset).

As shown in the scheme in Fig. 10, we train a feed-forward NN on the source dataset (Graphite electrode in the LIB electrode manufacturing experimental dataset and the GDL_200_ in the stochastically generated dataset) to predict a final property (density and mass loading in one case and geometric tortuosity in the other). This NN captures the variations in manufacturing conditions and the resulting changes in the target property. For the target dataset (Si-Gra and NMC on one side and GDL_1000_ on the other), we freeze each pre-trained NN (Fig. 10a) and add an extra layer to train and perform the same task (Fig. 10b). The new layer added for each transferred NN is an adapter, that processes the output data and extracts additional features specific to the different manufacturing conditions in the target domain. Therefore, adding this extra layer to accomodate different manufacturing conditions is similar to the TL parameter-based approach, previously discussed. It does not change the input features like feature-based TL or copy the weights like parameter-based TL. Instead, it uses the learned relationships in the pre-trained NN and adjust to the target property.

First, for the experimental dataset, two feed-forward NNs are trained using the Graphite source dataset (pre-trained NNs). One NN predicts the electrode density, and the other one predicts the electrode mass loading. Then, an additional layer is added to each of the pre-trained NNs, creating two TL-based NNs for each output target (electrode density and electrode mass loading). The NN architecture of all the four models are shown in Table 3, where the number of trainable parameters is also given. For both pre-trained and TL-based NNs we use 6 input parameters: AM weight percentage and solid content in the slurry formulation, coating speed and comma gap during slurry coating, and roll speed and roll gap during electrode calendering. Similarly, a feed-forward NN is trained using the GDL_200_ dataset to predict the geometric tortuosity by using as input parameters fiber diameter, fiber concentration, binder concentration, thickness, and compression factor. Then, an additional layer is added to this pre-trained NN to train the TL-based NN on the GDL_1000_ dataset. These other architectures are shown in Table 4. Each hidden layer in all six models is activated using the ReLU function. The codes to train these models were written in Python by using TensorFlow^71^, along with other common scientific computing libraries^72^. They were trained on a 13th Gen Intel(R) Core(TM) i7-13700H with 32 GB of RAM.

**Table 3:** Pre-trained and TL-based NN architectures for electrode density and mass loading prediction.

Neural Network	Target output	Number of hidden layers	Number of nodes per layer	Total number of trainable parameters
Pre-trained	Density	4	4	93
	Mass loading	3	3	49
TL-based	Density	1 (plus the frozen previous layers)	4	13
	Mass loading	1 (plus the frozen previous layers)	3	10

**Table 4:** Pre-trained and TL-based feed-forward NN architectures for the GDL geometric tortuosity prediction.

Neural Network	Number of hidden layers	Number of nodes per layer	Total number of trainable parameters
Pre-trained	3	3	46
TL-based	1 (plus the frozen previous layers)	3	10

## Supplementary information

Supplementary Information

## Data Availability

The datasets used and analysed during the current study are available from the corresponding author on reasonable request.

## Code availability

The underlying code for this study is not publicly available for proprietary reasons.
