# Supplementary material for: Transfer learning assessment of small datasets relating manufacturing parameters with electrochemical energy cell component properties
Source: NPJ Adv Manuf. 2025 Apr 18;2(1):14. doi: 10.1038/s44334-025-00024-1 (PMC12008025; doi:10.1038/s44334-025-00024-1)
Supplement: Supplementary file 1 — Supplementary Information [file 44334_2025_24_MOESM1_ESM.pdf]

Supporting Information

Transfer Learning Assessment of Small Datasets Relating Manufacturing Parameters with Electrochemical Energy Cell Component Properties

Francisco Fernandez[1,2], Soorya Saravanan[1,2], Rashen Lou Omongos [1,2], Javier F. Troncoso[1,2], Diego E. Galvez-Aranda[1,2], Alejandro A. Franco[1,2,3,4*]

[1] Laboratoire de Réactivité et Chimie des Solides (LRCS), Université de Picardie Jules Verne, Hub de l'Energie, UMR CNRS 7314,15 rue Baudelocque, 80039 Amiens, France

[2] Réseau sur le Stockage Electrochimique de l´Energie (RS2E), FR CNRS 3459, Hub de l'Energie, 15 rue Baudelocque, Amiens Cedex, 80039, France

[3] ALISTORE-European Research Institute, FR CNRS 3104, Hub de l'Energie, 15 rue Baudelocque, Amiens Cedex, 80039 France

[4] Institut Universitaire de France, 103 Boulevard Saint Michel, Paris, 75005, France

∗Corresponding author: alejandro.franco@u-picardie.fr

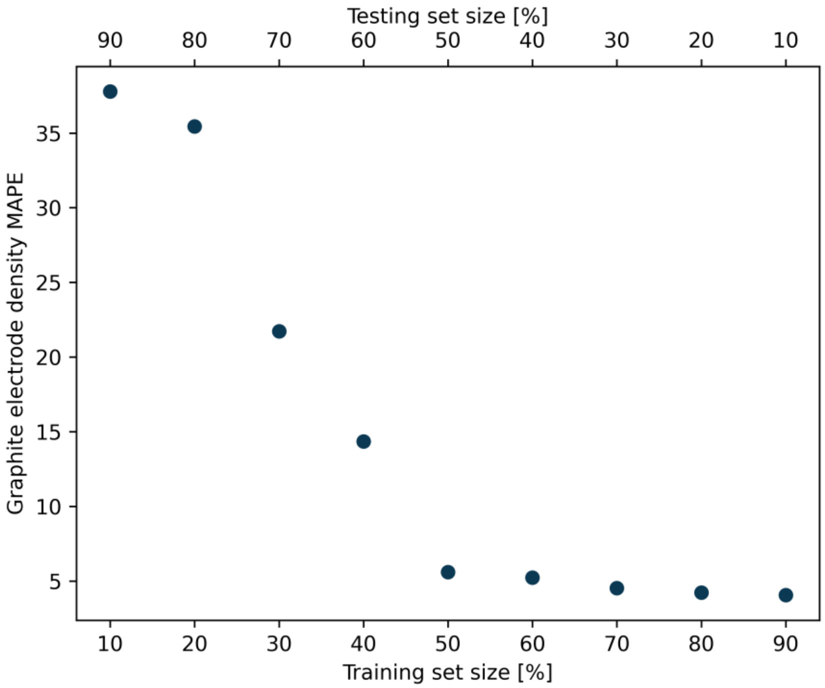

Figure S1. Sensitivity of Graphite electrode density MAPE at different training-testing split percentages.

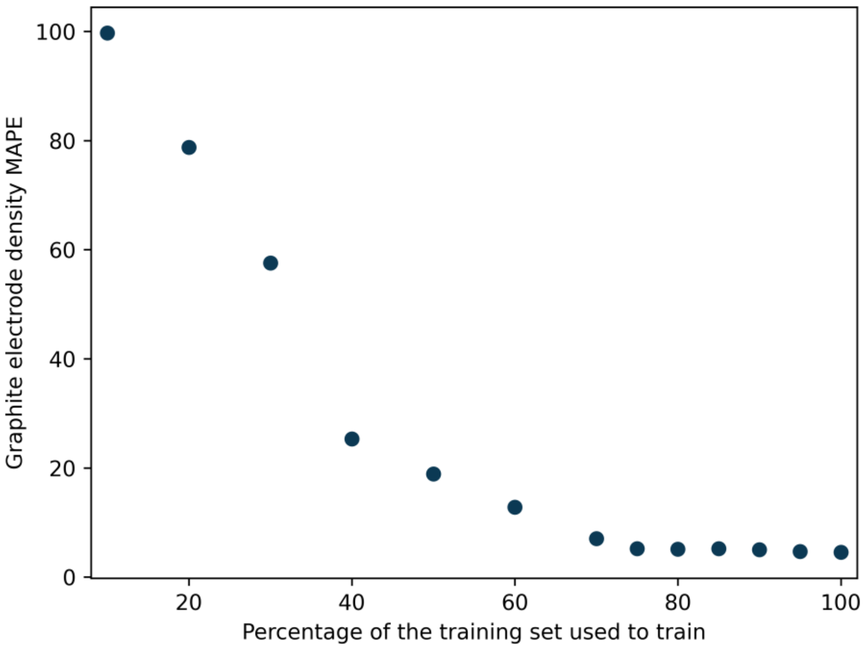

Figure S2. Ablation study for the Graphite electrode density MAPE of the NN trained on the Graphite vast dataset as

a function of the percentage of this dataset used for the training.

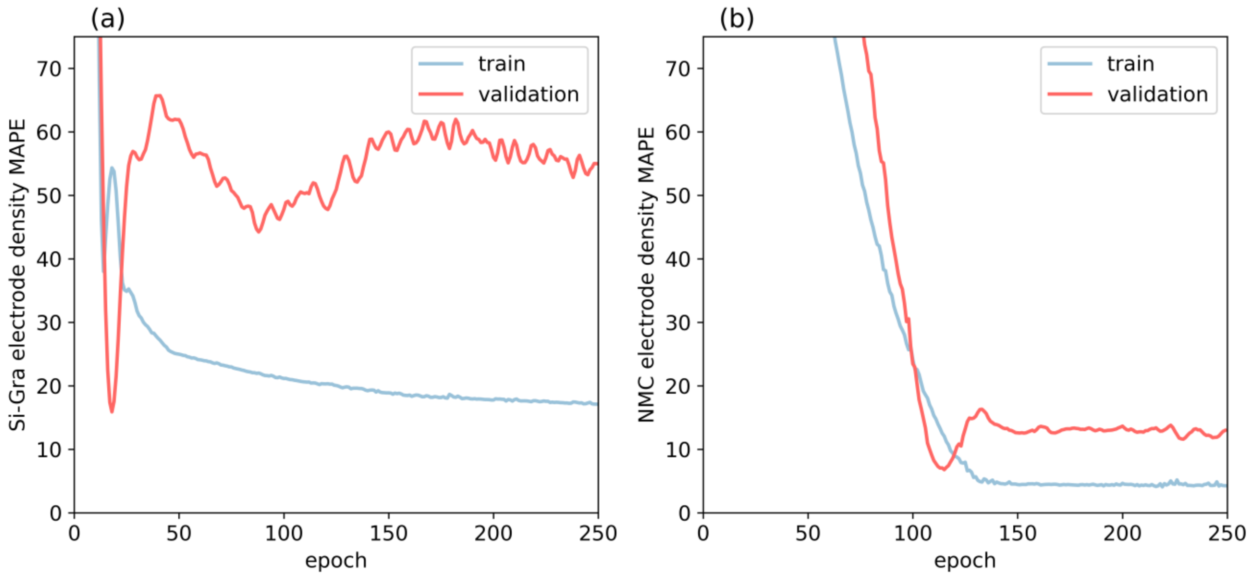

Figure S3. Electrode density MAPE loss plot of the NNs trained on small (a) Si-Gra and (b) NMC datasets.

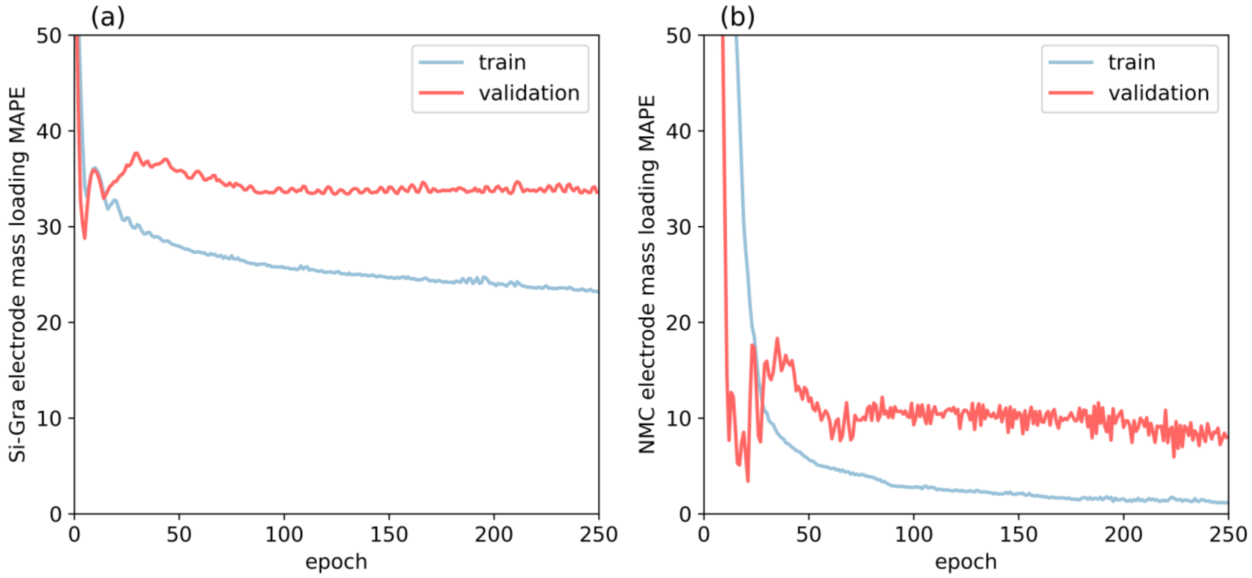

Figure S4. Electrode mass loading MAPE loss plot of the NNs trained on small (a) Si-Gra and (b) NMC datasets.

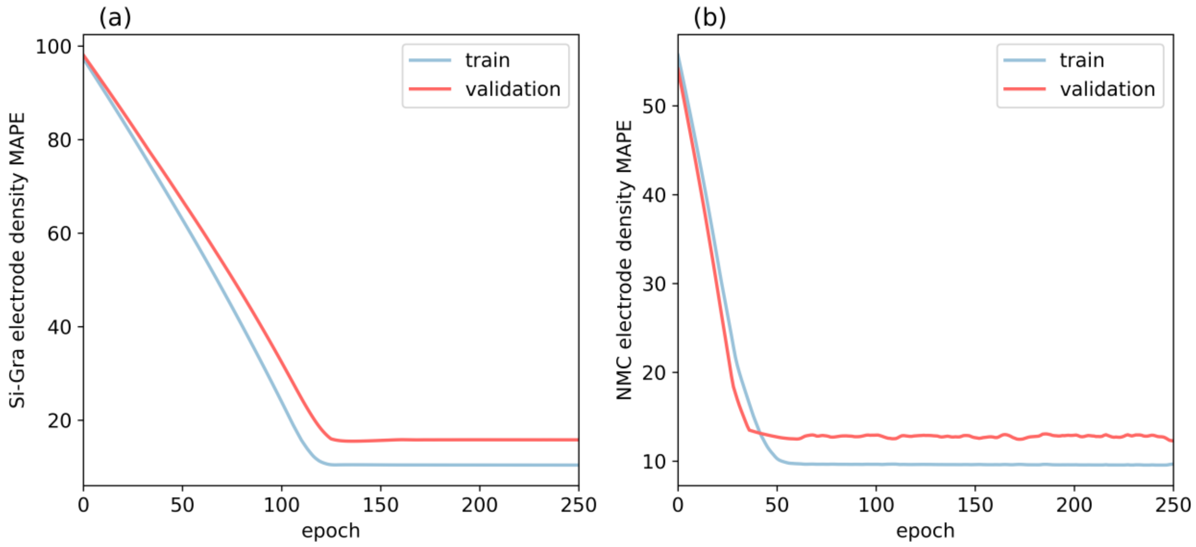

Figure S5. Electrode density MAPE loss plot for the TL-based NNs trained on small (a) Si-Gra and (b) NMC datasets.

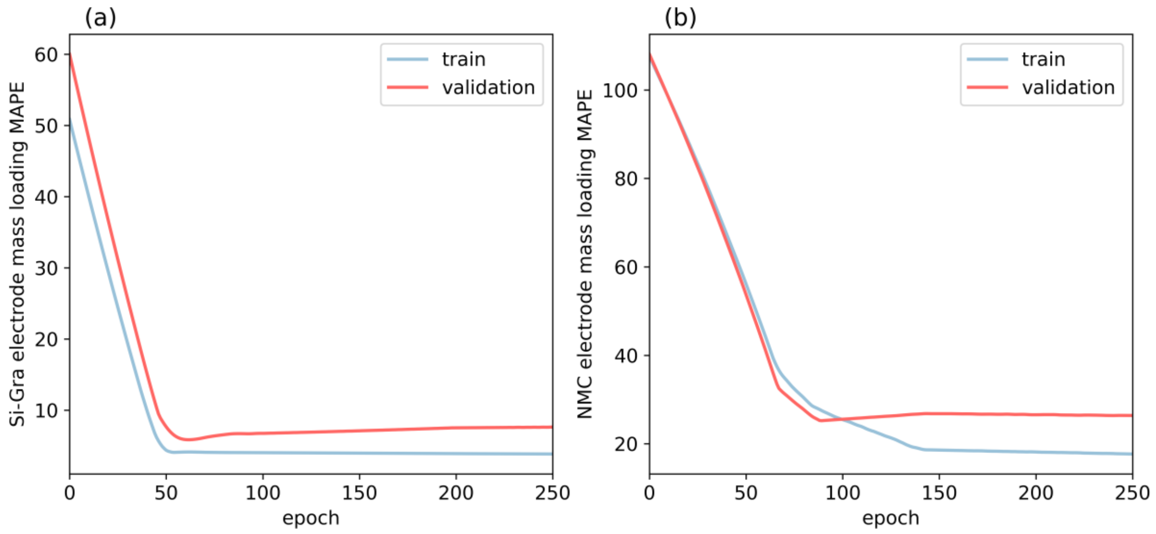

Figure S6. Electrode mass loading MAPE loss plot for the TL-based NNs trained on small (a) Si-Gra and (b) NMC datasets.

Table S1. MAPE comparison of both pre-trained and TL-based NNs with different Machine Learning baseline models for all the experimental (LIB electrode) datasets.

	Density MAPE [%]			Mass Loading MAPE [%]		
	Graphite	Si-Gra	NMC	Graphite	Si-Gra	NMC
Neural Network	4.53	14.53	8.09	13.90	3.71	10.61
Average Regressor	22.31	23.05	45.73	135.99	121.99	737.33
Linear Regressor	5.49	7.59	23.07	26.97	16.91	136.45
Random Forest	5.35	7.56	17.76	23.42	22.38	134.46

Table S2. Evaluation of the error of each one of the random selections of train/test split in the K-fold cross-validation for the pre-trained NNs.

K-fold cross-validation	Density MAPE	Mass loading MAPE	Geometric tortuosity MAPE
1	4.5	12.8	1.6
2	2.8	13.0	1.9
3	3.8	12.9	1.9
4	5.0	13.2	2.1
5	3.6	12.6	1.7

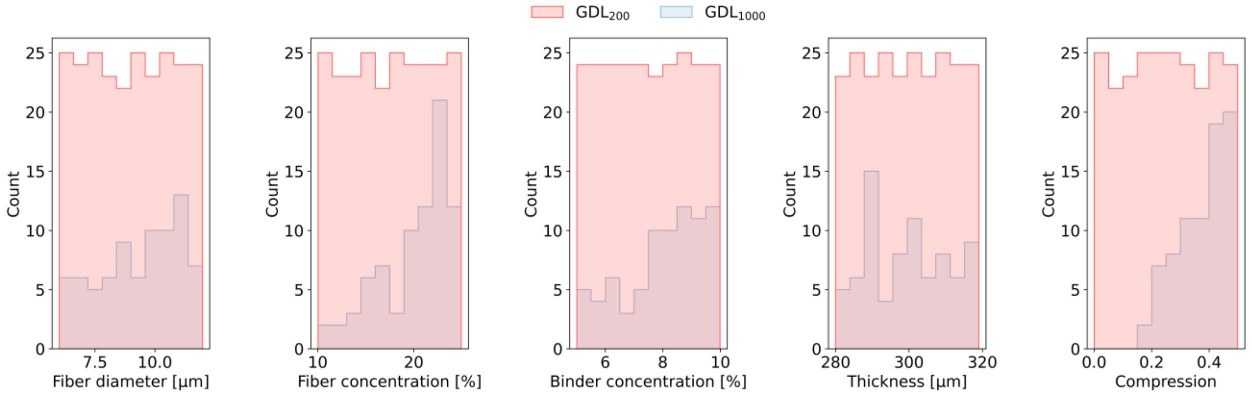

Figure S7. Distributions of the manufacturing parameters (in the stochastically generated GDL datasets) used as input features in the NNs for the GDL geometric tortuosity prediction: GDL_{200} (red), GDL_{1000} (blue).

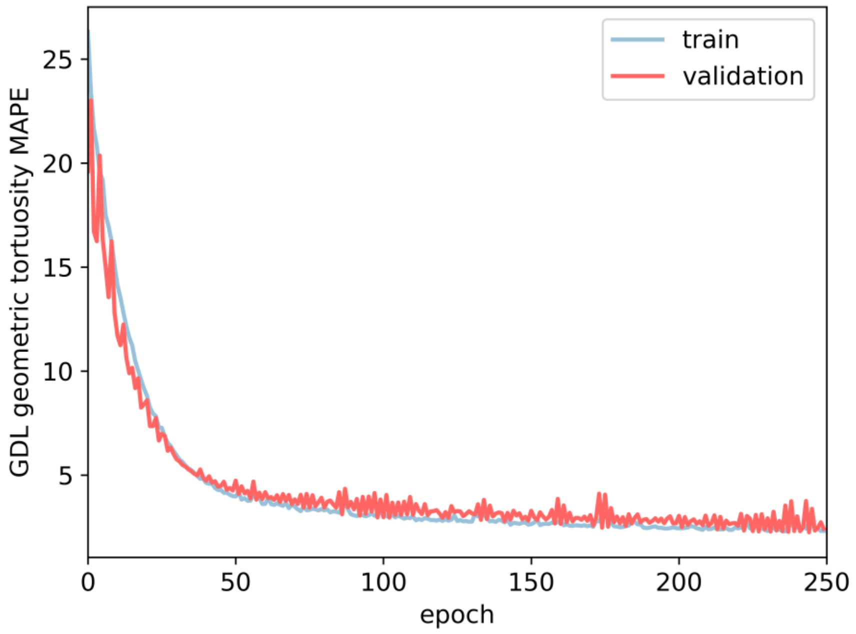

Figure S8. GDL geometric tortuosity MAPE loss plot for both training (blue curve) and validation (red curve) sets when the NN is trained on the GDL_{200} dataset.

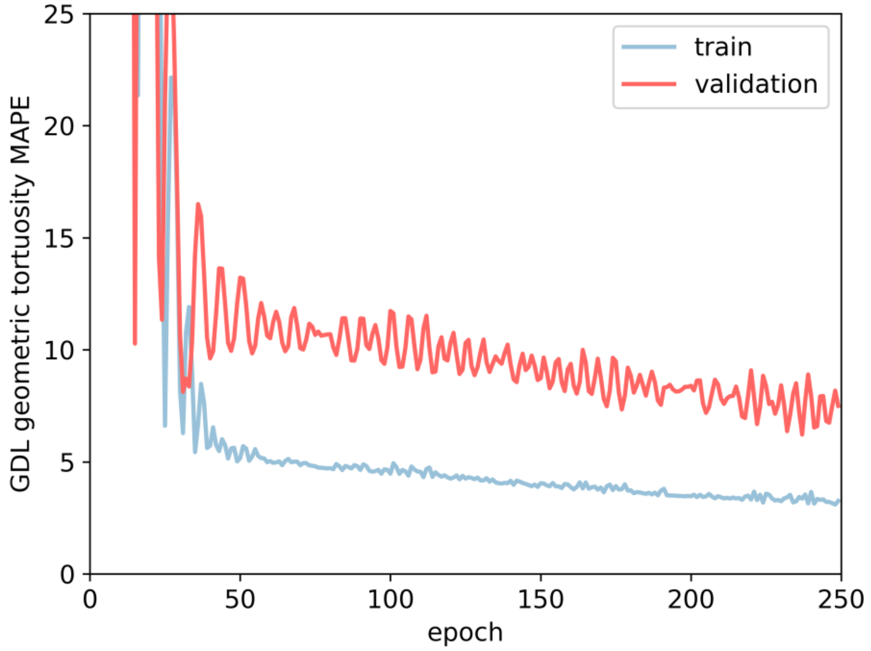

Figure S9. GDL geometric tortuosity MAPE loss plot for both training (blue curve) and validation (red curve) sets when the NN is trained on the small GDL_{1000} dataset.

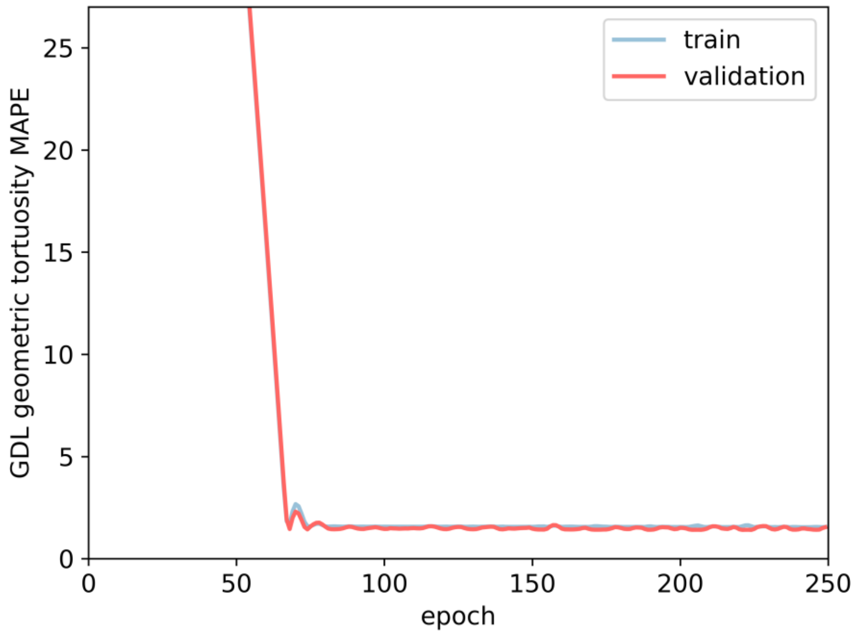

Figure S10. GDL geometric tortuosity MAPE loss plot for both train (blue curve) and validation (red curve) sets when the TL-based NN is trained on the small GDL_{1000} dataset.

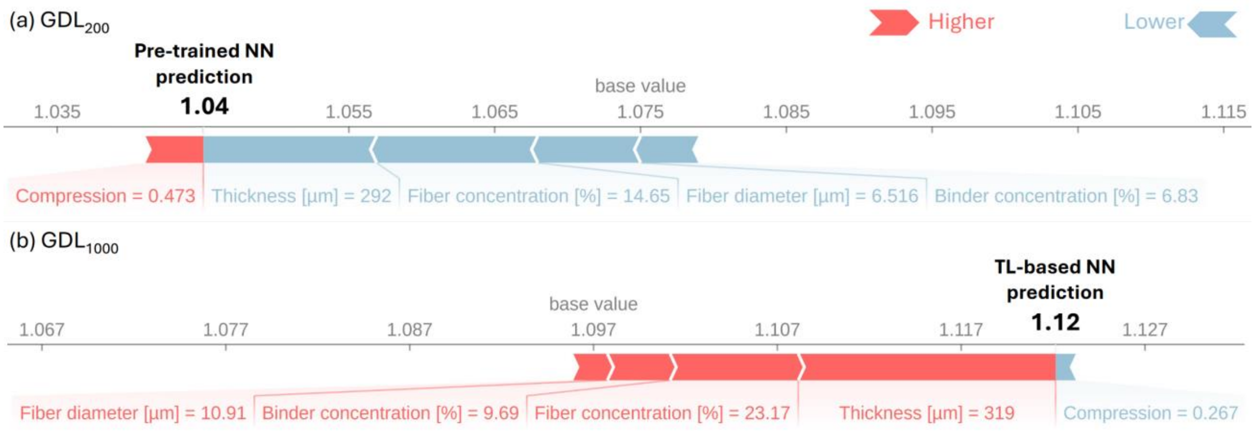

Figure S11. Representation of Shapley values for the NN prediction of the GDL geometric tortuosity for a randomly selected test microstructure from (a) GDL_{200} and (b) GDL_{1000} datasets.

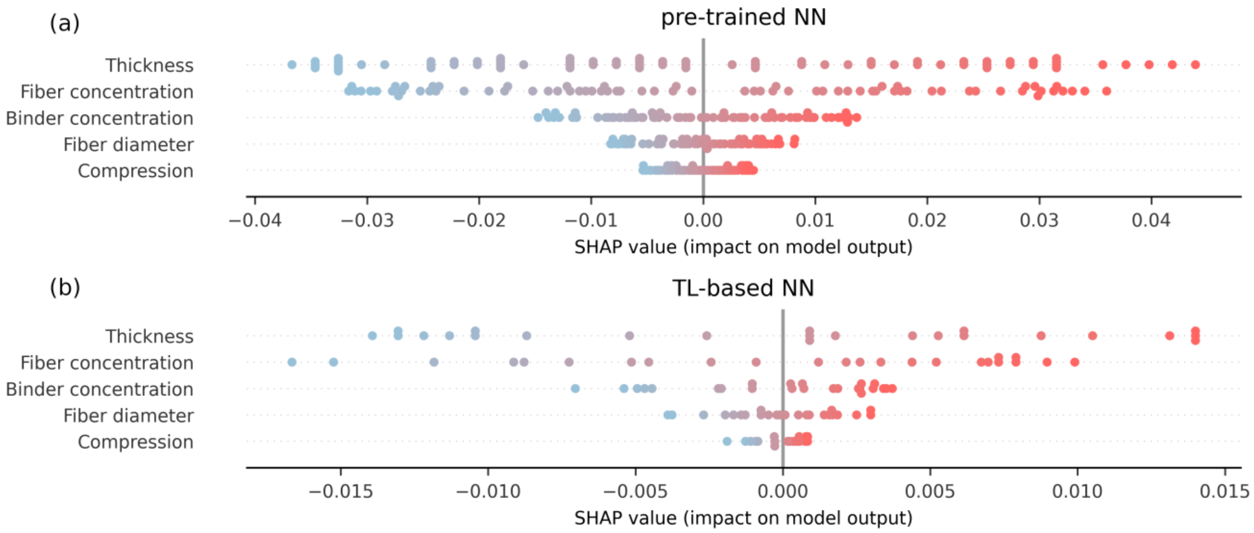

Figure S12. Global representation of Shapley values for the NNs predictions of the GDL geometric tortuosity for each microstructure in (a) GDL_{200} and (b) GDL_{1000} datasets.
